# Predicting response to CGRP-monoclonal antibodies in patients with migraine in Japan: a single-centre retrospective observational study

**DOI:** 10.1186/s10194-023-01556-7

**Published:** 2023-03-09

**Authors:** Keiko Ihara, Seiya Ohtani, Narumi Watanabe, Nobuyuki Takahashi, Naoki Miyazaki, Kei Ishizuchi, Satoko Hori, Ryo Takemura, Jin Nakahara, Tsubasa Takizawa

**Affiliations:** 1 Department of Neurology, Keio University School of Medicine, 35 Shinanomachi, Shinjuku-ku, Tokyo, 160-8582 Japan; 2 Division of Drug Informatics, Keio Univiersity Faculty of Pharmacy, 1-5-30 Shibakouen, Minato-ku, Tokyo, 105-8512 Japan; 3 Biostatistics Unit, Clinical and Translational Research Center, Keio University Hospital, 35 Shinanomachi, Shinjuku-ku, Tokyo, 160-8582 Japan

**Keywords:** Migraine, Japan, CGRPmAbs, Headache, Erenumab, Galcanezumab, Fremanezumab

## Abstract

**Background:**

Anti-calcitonin gene-related peptide monoclonal antibodies (CGRPmAbs) are a favourable option for patients with migraine who experience distressful headache disability and fail to respond to traditional preventive treatment options. However, since CGRPmAb has been available for only 2 years in Japan, the difference between good and poor responders remains unknown. We aimed to investigate the clinical characteristics of patients with migraine in Japan who responded well to CGRPmAb based on real-world data.

**Methods:**

We analysed patients who visited Keio University Hospital, Tokyo, Japan, between the 12^th^ of August 2021 and 31^st^ of August 2022, and were prescribed one of three CGRPmAbs (erenumab, galcanezumab, and fremanezumab) for more than 3 months. We recorded the patients’ basic migraine characteristics, such as pain quality, monthly migraine days (MMD)/monthly headache days (MHD), and the number of prior treatment failures. We defined good responders as patients whose MMDs decreased by more than 50% after 3 months of treatment and other patients as poor responders. We compared the baseline migraine characteristics between the two groups and performed logistic regression analysis based on the items that showed statistically significant differences.

**Results:**

In total, 101 patients were considered eligible for the responder analysis (galcanezumab: 57 (56%), fremanezumab: 31 (31%), and erenumab: 13 (13%)). After 3 months of treatment, 55 (54%) patients achieved ≥ 50% reduction in MMDs. Comparisons between ≥ 50% responders and non-responders revealed that age was significantly higher (*p* = 0.003), and MHD and total prior treatment failures were significantly lower (*p* = 0.027, 0.040, respectively), in responders than in non-responders. Age was a positive predictive factor, and the total number of prior treatment failures and past medical history of immuno-rheumatologic diseases were negative predictive factors of CGRPmAb responsiveness in Japanese patients with migraine.

**Conclusions:**

Patients with migraine who are older, with fewer prior treatment failures and no past history of immuno-rheumatologic disease, may respond well to CGRPmAbs.

## Background

Migraine is one of the most common neurological disorders, and places a significant burden on patients. The prevalence of migraine has been reported to be 14.4% worldwide [1] and 8.4% in Japan [2]. Migraine treatment options have been inefficient for decades; however, anti-calcitonin gene-related peptide monoclonal antibodies (CGRPmAbs) have recently drawn attention. Previous reports on clinical trials and real-world research show that CGRPmAbs produce substantially better outcomes than other treatments, without causing severe adverse effects [3–9]. However, CGRPmAbs also have disadvantages; they are more expensive, cause minor adverse effects such as injection site reactions and constipation, and are ineffective in some patients [10].

Currently, in Japan, CGRPmAbs (galcanezumab, fremanezumab, and erenumab) can be used for patients with ≥ 4 migraine days per month and for those who have experienced treatment ineffectiveness/intolerance, or have strong concerns about side effects, with at least one traditional migraine-preventive drug (i.e., lomerizine, propranolol, valproate) [10]. Regarding the expense of CGRPmAbs in Japan, the cost of CGRPmAbs is reimbursed as long as the criteria for approved indications of CGRPmAbs have been met. The co-payment is usually 30% of the total medical costs and may be partially or fully waived for elderly individuals, infants, and low-income patients. Thus, to optimise the use of CGRPmAbs, it would be ideal to predict the responsiveness of each patient before prescribing.

The literature to date has shown that the response to CGRPmAbs is positively associated with a lower number of failed preventative medications [11–14], unilateral pain localisation [13–15], better response to triptans [13, 16], lower number of monthly analgesic intakes at baseline [11, 17, 18], shorter duration of medication-overuse headache (MOH) [11, 13, 17, 19], and lower body mass index (BMI) [13], and negatively associated with the existence of psychiatric conditions [14, 20]. However, the results of these real-world studies have not always been consistent. For instance, baseline migraine frequency in good responders was higher in one study [14] but lower in another [21]. In addition, one study showed higher baseline Migraine Disability Assessment Scale (MIDAS) scores in good responders [12], whereas another revealed lower MIDAS scores in poor responders [11, 18].

These contradictory findings could be attributed to differences in the study population (i.e., the ratio of patients with episodic migraine to patients with chronic migraine), the definition of good responders, prescription guidelines, and insurance systems within each country. In order to appropriately prescribe CGRPmAbs to patients in Japan, real-world data on patients with migraine in this country are urgently required. To our knowledge, this is the first real-world study to investigate the clinical characteristics of good and poor responders among patients with migraine in Japan.

Importantly, migraines might have unique characteristics in Asian patients, given reports of lower overall prevalence [22, 23] and prevalence of migraine with aura [22] in Asian countries than in Western countries, as well as differences in characteristics (e.g. shorter duration [24] and the potential genetic basis of migraine [22, 25]. Moreover, Asians tend to have lower BMI compared to that in the Western population [26], which might affect the response to CGRPmAbs. Thus, analyzing patients with migraine specifically in Japan has added value and is the focus of this study.

## Methods

### Study design

We conducted a single-centre, retrospective, real-world study on patients with migraine who were treated with CGRPmAbs at Keio University Hospital in Tokyo, Japan. This study was approved by the Institutional Review Board of Keio University School of Medicine (approval number: 20211144). Patients were informed of this observational study via the institute’s website and could opt out of the study. The need for informed consent was waived by the Ethics Committee of the Keio University School of Medicine, in accordance with national regulations (Ethical Guidelines for Medical and Biological Research Involving Human Subjects). All methods were carried out in accordance with relevant guidelines and regulations. 

### Patients

**Fig. 1:**
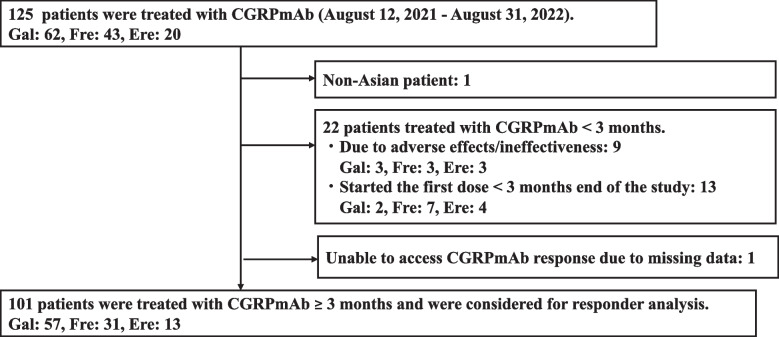
Study design Gal, Galcanezumab; Fre, Fremanezumab; Ere, Erenumab; CGRPmAb, anti-calcitonin gene-related peptide monoclonal antibody

The inclusion criteria for responder analysis were as follows: treatment of ≥ 3 months of galcanezumab (240 mg/120 mg/120 mg), erenumab (70 mg), or fremanezumab (225 mg monthly or 675 mg quarterly starting from 225 mg monthly) as their first CGRPmAb (de novo) from the Headache Group of Keio University Hospital; receipt of the first dose of CGRPmAb between the 12^th^ of August 2021 (when the drug became available at the hospital) and 31^st^ of August 2022; fulfilment of the diagnostic criteria for migraine (including probable migraine) according to the International Classification of Headache Disorders, 3^rd^ edition (ICHD-3); and age ≥ 18 years. The patients were diagnosed with migraine by a headache specialist (TT). Non-Asian patients were excluded (Fig. 1).

### Research items

We retrospectively collected demographic data (age, sex, height, and weight), medical history (psychiatric, gastrointestinal, vascular, hormonal, cancer, respiratory, and immuno-rheumatologic), and the following headache characteristics: onset age, family history of headache, migraine characteristics (unilateral pain, pulsating pain, or aggravation by routine physical activity), pain intensity (0–10; numerical rating scale [NRS]), associated symptoms (photophobia, phonophobia, and nausea/vomiting; none, mild, moderate, or severe), and the presence of aura. The headache specialist explained the criteria for migraine based on the ICHD-3 to all patients who were asked to track headache and migraine days (including probable migraine days). Patients completed a questionnaire on monthly migraine days (MMD), monthly headache days (MHD), monthly acute medication intake days (AMD) at baseline and MMD after 3 months of treatment. A month was defined as 28 days. The headache specialist verified the accuracy and reliability of the completed questionnaire by interviewing and occasionally reviewing each patient’s headache diary. Patients were classified as having episodic migraine or chronic migraine, according to the ICHD-3. Patients were also diagnosed with MOH based on the ICHD-3. Patients completed the Generalized Anxiety Disorder-7 (GAD-7) questionnaire [27, 28] and Patient Health Questionnaire-9 (PHQ-9) [29] upon CGRPmAb administration to determine the extent of anxiety and depression, respectively. We also collected patient migraine-preventive drug data, including failures of preventative drugs (lomerizine, propranolol, valproate, amitriptyline, or topiramate) before CGRPmAb treatment and response frequency to triptan (0, 1, 2, 3 out of three uses) [10].

### Assessments

We calculated the percentage reduction in MMD from baseline after 3 months of treatment. We assessed the characteristics of patients who responded with ≥ 25%, 50%, and 75% reduction in MMD by comparing the average scores between responders and non-responders for the following items: patient characteristics (age, onset age, sex, and body mass index [BMI]), diagnosis (episodic or chronic, presence of aura), migraine characteristics (unilateral/bilateral, pulsating/non-pulsating, pain intensity on NRS, duration, and aggravation by routine physical activity), MHD, MMD, AMD, whether patients had MOH or not, associated symptoms (photophobia, phonophobia, and nausea/vomiting), treatment (triptan response and total prior failures), other scores (GAD-7 and PHQ-9), medical history (psychiatric, gastrointestinal, vascular, hormonal, cancer, respiratory, or immuno-rheumatologic), and family history of headache. Triptan response was defined as how many times triptan successfully relieved a headache out of three usages.

### Statistical analysis

We compared average scores using the unpaired t-test for continuous variables and chi-square test for categorical variables. All two-sided *p*-values < 0.05 were considered statistically significant. We also used univariate logistic regression models to determine the baseline characteristics associated with a 50% response to CGRPmAb. The variables significantly associated with the response (*p* < 0.1) were then tested as independent variables in a multivariate logistic regression model to evaluate potentially independent associations with responder status and to check for collinearity. We reported the odds ratios (ORs) and 95% confidence intervals (CIs) for the risk factors. Missing data were excluded. Statistical analyses were performed using R version 4.0.3 [30].

## Results

### Participants’ demographics and baseline parameters

From the 12th of August 2021 to 31st of August 2022, 125 patients started CGRPmAb treatment at the Keio University Hospital Headache outpatient clinic (galcanezumab: 62 (50%), fremanezumab: 43 (34%), and erenumab: 20 (16%)) (Fig. 1). We excluded one non-Asian patient. In addition, we excluded two patients when analysing the triptan response, as they had not taken the medication multiple times. One patient did not complete PHQ-9 questionnaire and was excluded when analyzing PHQ-9 scores. Twenty-two patients received CGRPmAbs treatment for less than 3 months: 9 discontinued CGRPmAb due to adverse effects (light-headedness, hair loss, eczema, palpitation, throat itching, or constipation) or ineffectiveness, and 13 started CGRPmAbs later than 3 months before the end of the study period. We excluded one patient whose CGRPmAb response could not be assessed due to missing data. No patient was lost to follow-up. One-hundred-and-one patients were considered eligible for the responder analysis (galcanezumab: 57 (56%), fremanezumab: 31 (31%), and erenumab: 13 (13%)). In Japan, one of three CGRPmAbs can be selected after physician–patient discussions. Although erenumab was the first to be placed on the market globally, galcanezumab was launched first in Japan; accordingly, galcanezumab was the most frequently prescribed CGRPmAb in the present study. Additionally, many patients appreciated the convenience of fremanezumab, which can be administered quarterly, not necessarily monthly, making erenumab the least frequently prescribed CGRPmAb during the study period (self-injection of fremanezumab or erenumab was not available during the study period).

After 3 months of treatment, among 101 patients, 71 (70%), 55 (54%), and 31 (31%) patients achieved ≥ 25%, 50%, and 75% reduction in MMDs, respectively. The comparison of ≥ 25%, 50%, and 75% responders and non-responders revealed statistically significant differences in duration, photophobia, total prior treatment failures (*p* = 0.004, 0.012 and 0.023), age, MHD, and total prior treatment failures (*p* = 0.003 0.027, and 0.040), and triptan response and total prior treatment failures (*p* = 0.047 and 0.022), respectively (Table 1). Seven patients had a medical history of immuno-rheumatologic diseases: 1 was a ≥ 50% responder (with a history of Sjogren’s syndrome), and 6 were non-responders with the following histories: rheumatoid arthritis (*n* = 3), myasthenia gravis (*n* = 1), Sjogren’s syndrome (*n* = 1), and peripheral spondyloarthritis (*n* = 1).

**Table 1:** Demographic and clinical characteristics of responders and non-responders

		Average	≧ 25% response			≧ 50% response			≧ 75% response		
		All (101)	Responder (71)	Non-responder (30)	*P* value	Responder (55)	Non-responder (46)	*P* value	Responder (31)	Non-responder (70)	*P* value
Patient characteristics	Age	46.77 ± 1.29	47.87 ± 1.48	44.17 ± 2.53	0.189	**50.16 ± 1.53**	**42.72 ± 2.01**	**0.003**	49.06 ± 1.64	45.76 ± 1.7	0.237
	Onset age	21.9 ± 1.22	21.99 ± 1.44	21.7 ± 2.3	0.915	23.07 ± 1.74	20.5 ± 1.67	0.295	25.39 ± 2.15	20.36 ± 1.45	0.056
	Sex	83, 82%	59, 83%	24, 80%	0.930	47, 85%	36, 78%	0.497	25, 81%	58, 83%	1.000
	BMI	21.7 ± 0.39	21.41 ± 0.47	22.38 ± 0.71	0.261	21.83 ± 0.57	21.55 ± 0.53	0.720	22.33 ± 0.86	21.42 ± 0.42	0.283
Migriane characteristics	Chronic Migraine	44, 44%	28, 39%	16, 53%	0.286	20, 36%	24, 52%	0.163	13, 42%	31, 44%	0.998
	Aura	26, 26%	16, 23%	10, 33%	0.376	14, 25%	12, 26%	1.000	7, 23%	19, 27%	0.813
	Unilateral pain	72, 71%	50, 70%	22, 73%	0.956	38, 69%	34, 74%	0.755	18, 58%	54, 77%	0.086
	Pulsating pain	64, 63%	45, 63%	19, 63%	1.000	35, 64%	29, 63%	1.000	17, 55%	47, 67%	0.337
	Pain severity	6.07 ± 0.15	6.07 ± 0.18	6.07 ± 0.29	0.991	6.07 ± 0.21	6.07 ± 0.23	0.981	6.1 ± 0.3	6.06 ± 0.18	0.905
	Duration	5.12 ± 0.77	**3.68 ± 0.47**	**8.5 ± 2.22**	**0.004**	3.78 ± 0.59	6.71 ± 1.5	0.057	3.87 ± 0.77	5.66 ± 1.04	0.287
	Aggravation by routine physical activity	82, 81%	60, 85%	22, 73%	0.301	45, 82%	37, 80%	1.000	28, 90%	54, 77%	0.198
	MHD	14.96 ± 0.76	14.00 ± 0.88	17.23 ± 1.45	0.052	**13.42 ± 0.99**	**16.8 ± 1.13**	**0.027**	13.5 ± 1.34	15.6 ± 0.92	0.204
	MMD	12.5 ± 0.74	11.63 ± 0.82	14.58 ± 1.53	0.069	11.5 ± 0.95	13.71 ± 1.16	0.138	12.23 ± 1.3	12.63 ± 0.91	0.806
	AMD	9.7 ± 0.71	9.67 ± 0.78	9.78 ± 1.56	0.946	9.35 ± 0.85	10.13 ± 1.2	0.590	9.47 ± 1.03	9.81 ± 0.93	0.829
	MOH	28, 28%	17, 24%	11, 37%	0.288	12, 22%	16, 35%	0.220	9, 29%	19, 27%	1.000
Associated symptoms	Photophobia—none	22, 22%	**10, 14%**	**12, 40%**	**0.012**	9, 16%	13, 28%	0.186	5, 16%	17, 24%	0.497
	- mild	30, 30%	**26, 37%**	**4, 13%**		18, 33%	12, 26%		8, 26%	22, 31%	
	- moderate	32, 32%	**24, 34%**	**8, 27%**		21, 38%	11, 24%		13, 42%	19, 27%	
	- severe	17, 17%	**11, 15%**	**6, 20%**		7, 13%	10, 22%		5, 16%	12, 17%	
	Phonophobia—none	22, 22%	12, 17%	10, 33%	0.337	10, 18%	12, 26%	0.798	7, 23%	15, 21%	0.618
	- mild	35, 35%	26, 37%	9, 30%		20, 36%	15, 33%		8, 26%	27, 39%	
	- moderate	29, 29%	22, 31%	7, 23%		16, 29%	13, 28%		11, 35%	18, 26%	
	- severe	15, 15%	11, 15%	4, 13%		9, 16%	6, 13%		5, 16%	10, 14%	
	Nausea/vomitting—none	26, 26%	18, 25%	8, 27%	0.944	15, 27%	11, 24%	0.268	7, 23%	19, 27%	0.062
	- mild	41, 41%	28, 39%	13, 43%		18, 33%	23, 50%		8, 26%	33, 47%	
	- moderate	29, 29%	21, 30%	8, 27%		18, 33%	11, 24%		13, 42%	16, 23%	
	- severe	5, 5%	4, 6%	1, 3%		4, 7%	1, 2%		3, 10%	2, 3%	
Treatment	Triptan response (/3 times)—0	14, 14%	6, 8%	8, 29%	0.082	4, 7%	10, 23%	0.096	**2, 6%**	**12, 18%**	**0.047**
	- 1	26, 26%	20, 28%	6, 21%		13, 24%	13, 30%		**5, 16%**	**21, 31%**	
	- 2	26, 26%	20, 28%	6, 21%		17, 31%	9, 20%		**13, 42%**	**13, 19%**	
	- 3	33, 33%	25, 35%	8, 29%		21, 38%	12, 27%		**11, 35%**	**22, 32%**	
	Total prior treatment failures	1.82 ± 0.11	**1.66 ± 0.12**	**2.2 ± 0.23**	**0.023**	**1.62 ± 0.14**	**2.07 ± 0.17**	**0.040**	**1.45 ± 0.18**	**1.99 ± 0.13**	**0.022**
Other scores	GAD-7	4.92 ± 0.39	4.58 ± 0.44	5.73 ± 0.81	0.179	4.82 ± 0.54	5.04 ± 0.57	0.776	5.1 ± 0.7	4.84 ± 0.48	0.767
	PHQ-9	5.86 ± 0.42	5.76 ± 0.46	6.1 ± 0.89	0.709	6.07 ± 0.56	5.61 ± 0.64	0.582	6.57 ± 0.7	5.56 ± 0.52	0.271
Medical history	Psychiatric	27, 27%	17, 24%	10, 33%	0.466	14, 25%	13, 28%	0.927	8, 26%	19, 27%	1.000
	Gastrointestinal	26, 26%	16, 23%	10, 33%	0.376	14, 25%	12, 26%	1.000	6, 19%	20, 29%	0.465
	Vascular	8, 8%	3, 4%	5, 17%	0.087	3, 5%	5, 11%	0.526	2, 6%	6, 9%	1.000
	Hormonal	8, 8%	6, 8%	2, 7%	1.000	5, 9%	3, 7%	0.915	3, 10%	5, 7%	0.972
	Cancer	11, 11%	7, 10%	4, 13%	0.871	6, 11%	5, 11%	1.000	4, 13%	7, 10%	0.932
	Respiratory	11, 11%	9, 13%	2, 7%	0.592	7, 13%	4, 9%	0.744	4, 13%	7, 10%	0.932
	Immuno-rheumatologic	7, 7%	4, 6%	3, 10%	0.718	1, 2%	6, 13%	0.069	1, 3%	6, 9%	0.582
Family history of headache		58, 57%	38, 54%	20, 67%	0.317	27, 49%	31, 67%	0.099	13, 42%	45, 64%	0.061

*BMI* Body mass index, *MHD* Monthly headache days, *MMD* Monthly migraine days, *GAD-7* Generalized anxiety disorder-7, *PHQ-9* Patient health questionnaire-9, *AMD* Monthly acute medication intake days, *MOH* Medication-overuse headache

### Logistic regression analysis

Baseline characteristics were analysed using univariate and multivariate logistic regression models to screen for and identify the prognostic factors of ≥ 50% response to CGRPmAb. Univariate analysis revealed positive associations with age, response to triptans in 2–3 of 3 usages, and moderate photophobia, and negative associations with duration, MHD, prior treatment failures, medical history (immuno-rheumatologic), and family history of headaches (*p* < 0.1). Multivariate analysis revealed that age was a positive predictor of response, and total prior treatment failures and immuno-rheumatologic medical history were negative predictors of response, with significance (OR = 1.072, 0.512, and 0.027; CI = 1.025–1.121, 0.290–0.904, and 0.002–0.422; *p* = 0.002, 0.021, and 0.010, respectively) (Table 2).

**Table 2:** Univariate and multivariate analyses of determinants of ≥ 50% response

		Univariate		Multivariate	
		OR (95% CI)	*P* Value	OR (95% CI)	*P* Value
Patient characteristics	Age	**1.050 (1.015 ~ 1.087)**	**0.005**	**1.072 (1.025 ~ 1.121)**	**0.002**
	Onset age	1.018 (0.985 ~ 1.052)	0.292		
	Sex	1.632 (0.585 ~ 4.553)	0.350		
	BMI	1.019 (0.921 ~ 1.128)	0.717		
Migriane characteristics	Chronic Migraine	0.524 (0.236 ~ 1.163)	0.112		
	Aura	0.967 (0.395 ~ 2.368)	0.942		
	Unilateral pain	0.789 (0.33 ~ 1.886)	0.594		
	Pulsating pain	1.026 (0.455 ~ 2.312)	0.951		
	Pain severity	1.003 (0.775 ~ 1.299)	0.980		
	Duration	**0.935 (0.866 ~ 1.01)**	**0.086**	0.965 (0.871 ~ 1.070)	0.502
	Aggravation by routine physical activity	1.095 (0.403 ~ 2.976)	0.859		
	MHD	**0.942 (0.893 ~ 0.994)**	**0.029**	0.948 (0.884 ~ 1.017)	0.137
	MMD	0.96 (0.91 ~ 1.013)	0.139		
	AMD	0.985 (0.932 ~ 1.04)	0.586		
	MOH	0.523 (0.217 ~ 1.264)	0.150		
Associated symptoms	Photophobia—none				
	- mild	2.167 (0.706 ~ 6.645)	0.176	1.813 (0.442 ~ 7.439)	0.409
	- moderate	**2.758 (0.900 ~ 8.453)**	**0.076**	3.098 (0.754 ~ 12.726)	0.117
	- severe	1.011 (0.279 ~ 3.66)	0.987	1.233 (0.235 ~ 6.466)	0.804
	Phonophobia—none				
	- mild	1.6 (0.547 ~ 4.681)	0.391		
	- moderate	1.477 (0.485 ~ 4.497)	0.492		
	- severe	1.8 (0.476 ~ 6.813)	0.387		
	Nausea/vomitting—none				
	- mild	0.574 (0.213 ~ 1.549)	0.273		
	- moderate	1.2 (0.407 ~ 3.536)	0.741		
	- severe	2.933 (0.287 ~ 30.007)	0.364		
Treatment	Triptan response (/3 times)—0				
	- 1	2.5 (0.622 ~ 10.049)	0.197	2.133 (0.410 ~ 11.083)	0.368
	- 2	**4.722 (1.149 ~ 19.407)**	**0.031**	3.221 (0.585 ~ 17.718)	0.179
	- 3	**4.375 (1.124 ~ 17.033)**	**0.033**	1.340 (0.260 ~ 6.918)	0.727
	Total prior treatment failures	**0.675 (0.46 ~ 0.99)**	**0.044**	**0.512 (0.290 ~ 0.904)**	**0.021**
Other scores	GAD-7	0.986 (0.892 ~ 1.089)	0.774		
	PHQ-9	1.028 (0.934 ~ 1.131)	0.579		
Medical history	Psychiatric	0.867 (0.358 ~ 2.096)	0.751		
	Gastrointestinal	0.967 (0.395 ~ 2.368)	0.942		
	Vascular	0.473 (0.107 ~ 2.097)	0.324		
	Hormonal	1.433 (0.324 ~ 6.349)	0.635		
	Cancer	1.004 (0.286 ~ 3.531)	0.995		
	Respiratory	1.531 (0.419 ~ 5.598)	0.519		
	Immuno-rheumatologic	**0.123 (0.014 ~ 1.066)**	**0.057**	**0.027 (0.002 ~ 0.422)**	**0.010**
Family history of headache		**0.467 (0.207 ~ 1.051)**	**0.066**	0.577 (0.196 ~ 1.700)	0.318

*OR* Odds ratio, *CI* Confidence interval

## Discussion

Our results suggest that ≥ 50% CGRPmAb response is significantly associated with older age, fewer MHD, and fewer prior treatment failures, and can be predicted based on age, total prior treatment failures, and immuno-rheumatologic medical history. Several parameters that are reportedly associated with response, such as medication overuse [11, 17, 19], unilateral pain localisation [15], medical history of psychiatric disease [14, 20], and MMD [18], were not significantly different between responders and non-responders in the present study.

The responders were older, and age was positively associated with ≥ 50% response in the univariate logistic analysis. Although age could have been correlated with other factors that could affect the response to CGRPmAb, it was significantly positively associated with response in the multivariate logistic analysis. However, this result should be considered carefully because the sample size was small, and age was reported as a negative predictor of response in a previous real-world study, contrary to our results [17].

The number of total prior treatment failures was negatively associated with response in all responder rates (25%, 50%, and 75%) and has been reported as a negative predictor of response in multiple previous reports [11, 12]. This could suggest the robustness of the association between the number of total prior treatment failures and the CGRPmAb response.

Although the number of prior treatment failures as a response predictor has been reported in previous studies, this parameter has the following limitations. Firstly, the definition of failure varies among studies and is often not clearly specified. Secondly, because it is not always easy to separate discontinuation due to adverse effects from discontinuation due to ineffectiveness, prior treatment failures often include discontinuation due to adverse effects. Thirdly, multiple failures of preventive treatments could suggest that the patient’s headache was not a typical migraine and may indicate other diseases or pathophysiologies. Finally, in some countries, the number of prior treatment failures is included in the criteria for using CGRPmAb under health insurance, which could have caused a bias in the results of previous real-world studies.

The existence of immuno-rheumatologic comorbidities negatively affected the CGRPmAb response. This could be attributed to the bidirectional association between migraine and rheumatoid arthritis, the potentially increased risk of MOH, and the use of biological agents.

Previous studies reported that migraine increases the risk of rheumatoid arthritis [31, 32]. One study from Korea reported that the adjusted hazard ratio for rheumatoid arthritis in the migraine without aura group was 1.48 [31]. Another study reported that the crude hazard ratio for rheumatoid arthritis in the migraine group was 2.15 [32]. In addition, rheumatoid arthritis is associated with an increased risk of migraine, as the adjusted hazard ratio for migraine without aura in the rheumatoid arthritis group was reported as 1.35 [31].

Four of the six non-responders with immuno-rheumatologic diseases in this cohort were diagnosed with MOH. Although MOH was not a negative predictor in our study, it was previously reported in poor responders, possibly inhibiting their response to CGRPmAb [11, 17, 19].

Four non-responders were prescribed intravenous immunoglobulin (IVIg) or biologic agents for immuno-rheumatologic disease: IVIg for myasthenia gravis, infliximab or tocilizumab for rheumatoid arthritis, and golimumab for peripheral spondyloarthritis. These agents could have affected the effects of CGRPmAbs; thus, drug-drug interactions in non-responders should be considered in further studies.

This is the first real-world study from Japan that described the characteristics of CGRPmAb responders and predictive factors of response in a real-world setting. Since this study was conducted in a university hospital with many departments, the majority of patients had various other medical histories in addition to migraine, which enriched our analyses on the association between past medical histories and CGRPmAb response. Differences between the present and previous studies may be attributable to differences in the genetic background of migraine, the difference in the guidelines for CGRPmAbs prescription, and Japan’s wide insurance coverage for CGRPmAbs, which enabled the inclusion of patients with less severe migraine in this study.

However, this study has some limitations, including its small sample size, retrospective nature, single-centre design, and short observation period. In addition, the primary endpoint (MMD) was mainly assessed with questionnaires and not by actual headache diaries, which were only checked in some cases. Due to potential recall bias, this approach could have less accuracy compared to those used in other clinical trials equipped with electronic diaries. In addition, we excluded those patients who did not continue using CGRPmAbs for three months. This could have caused a selection bias in our study. Thus, further research is necessary to elucidate the effects of CGRPmAbs in the Japanese population.

## Conclusions

Our single-centre observational retrospective study suggests that CGRPmAb response can be predicted based on age, total number of prior treatment failures, and immuno-rheumatologic medical history. Our results partly differ from those in other countries, suggesting the importance of real-world CGRPmAb studies in Asian population.

## Data Availability

The datasets analysed during the current study are available from the corresponding author on reasonable requests.

## Abbreviations

CGRPmAbs: Anti-calcitonin gene-related peptide monoclonal antibodies
MMD: Monthly migraine days
MHD: Monthly headache days
BMI: Body mass index
ICHD-3: International Classification of Headache Disorders 3^rd^ edition
AMD: Monthly acute medication days
NRS: Numerical rating scale
MOH: Medication-overuse headache
OR: Odds ratio
CI: Confidence interval
MIDAS: Migraine Disability Assessment Scale
GAD-7: Generalized Anxiety Disorder-7
PHQ-9: Patient Health Questionnaire-9
IVIg: Intravenous immunoglobulin
